# Acute Phase Proteins as Early Predictors for Immunotherapy Response in Advanced NSCLC: An Explorative Study

**DOI:** 10.3389/fonc.2022.772076

**Published:** 2022-01-31

**Authors:** Marc A. Schneider, Adriana Rozy, Sabine Wrenger, Petros Christopoulos, Thomas Muley, Michael Thomas, Michael Meister, Tobias Welte, Joanna Chorostowska-Wynimko, Sabina Janciauskiene

**Affiliations:** ^1^ Translational Research Unit, Thoraxklinik at Heidelberg University Hospital, Heidelberg, Germany; ^2^ Translational Research Center Heidelberg (TLRC), Member of the German Center for Lung Research (DZL), Heidelberg, Germany; ^3^ Laboratory of Molecular Diagnostics and Immunology, National Institute of Tuberculosis and Lung Diseases, Warsaw, Poland; ^4^ Department of Respiratory Medicine, Hannover Medical School, Hannover, Germany; ^5^ Biomedical Research in End Stage and Obstructive Lung Disease Hannover (BREATH), Member of the German Center for Lung Research (DZL), Hannover, Germany; ^6^ Department of Thoracic Oncology, Thoraxklinik at University Hospital Heidelberg, Heidelberg, Germany

**Keywords:** NSCLC, checkpoint inhibitors, immunotherapy, acute phase proteins, progression-free survival

## Abstract

In the last decade, targeting the immune system became a promising therapy in advanced lung cancer stages. However, in a clinical follow-up, patient responses to immune checkpoint inhibitors widely differ. Peripheral blood is a minimally invasive source of potential biomarkers to explain these differences. We blindly analyzed serum samples from 139 patients with non-small cell lung cancer prior to anti-PD-1 or anti-PD-L1 therapies to assess whether baseline levels of albumin (ALB), alpha-1 acid glycoprotein (AGP), alpha1-antitrypsin (AAT), alpha2-macroglobulin (A2M), ceruloplasmin (CP), haptoglobin (HP), alpha1-antichymotrypsin (ACT), serum amyloid A (SAA), and high-sensitivity C-reactive protein (hs-CRP), have a predictive value for immunotherapy success. Disease progression-free survival (PFS) was calculated based on RECIST 1.1 criteria. A multivariate Cox regression analysis, including serum levels of acute-phase proteins and clinical parameters, revealed that higher pre-therapeutic levels of HP and CP are independent predictors of a worse PFS. Moreover, a combined panel of HP and CP stratified patients into subgroups. We propose to test this panel as a putative biomarker for assessing the success of immunotherapy in patients with NSCLC.

## Introduction

Programmed death ligand-1 (PD-L1) is a transmembrane protein induced by pro-inflammatory substances in many cell types, including cancer cells, and acts as a suppressor of immune response when bound to its complementary ligands. PD-L1 combines with programmed death (PD-1) receptor and inhibits immune cell activation (1). Checkpoint-inhibitor blockade is based on highly selective humanized monoclonal antibodies against PD-1 or PD-L1, helping the host immune system to identify and destroy tumour cells. Antibodies targeting the PD1-PDL1 axis are used both as a first- or second-line therapy for many malignancies, including lung cancers (2). The PD-1 inhibitors nivolumab and pembrolizumab, and PD-L1 inhibitors atezolizumab and durvalumab are currently considered as the breakthroughs and the most successful therapies for advanced non-small cell lung cancer (NSCLC). When assessed in different settings, these drugs seem to improve survival rates and show less severe toxicity than chemotherapy (3). Studies comparing survival benefits between standard platinum-based chemotherapy versus immunotherapy have shown significant benefit in the selected population of NSCLC patients both as the first (pembrolizumab, atezolizumab) or second-line therapies (4–6).

Despite certain advantages, immunotherapy does not show an expected benefit for all patients (7, 8). It seems that only about 20-25% of NSCLC patients positively respond to immunotherapy (9). Therefore, further studies are necessary to identify patients who may benefit from immune checkpoint blockade therapy (10) and to determine an appropriate sequence and/or combination of chemotherapy with immunotherapy. Concurrently, effective predictive biomarkers are necessary to enable personalized therapy and to guide designs of clinical trials.

In advanced NSCLC patients, evaluation of PD-L1 expression by immunohistochemistry is used as the primary biomarker for selecting patients as eligible for receiving anti-PD-L1 therapy. Yet, this assay is not able to identify conclusively non-benefiting patients (11). Likewise, other researched biomarker candidates have not been proven to be helpful in clinical settings. Only in a small subset of cancers mismatch repair /microsatellite instability and tumour mutations can serve as biomarkers for predicting immune checkpoint inhibitors efficacy (12).

The concentrations of plasma acute phase proteins (APPs) are changing during lung cancer development (13), and although these proteins are nonspecific inflammatory markers, they might be useful as biomarkers for disease management and prognosis. For example, elevated pre-operative levels of C-reactive protein (CRP) have been associated with inability to achieve complete resection in patients with NSCLC (14–16). Independently of tumour stage, patients with higher plasma CRP levels were found to show significantly lower overall survival than those with lower levels of CRP (15, 17). Higher serum levels of amyloid A (SAA) have also been found in patients with NSCLC as compared to healthy controls (18–22). Cho et al. reported that patients with a survival ≥ 5 years have significantly lower SAA than patients with a survival < 5 years (23). To date, various reports suggest that APPs have a profound impact on cancer development and the body’s innate immune system, however a putative prognostic value of combined serum APPs in NSCLC patients treated with immune therapy has not been explored.

We present data on serum levels of albumin (ALB), alpha-1 acid glycoprotein (AGP), alpha1-antitrypsin (AAT), alpha2-macroglobulin (A2M), ceruloplasmin (CP), haptoglobin (HP), alpha1-antichymotrypsin (ACT), serum amyloid A (SAA), and high-sensitivity C-reactive protein (hs-CRP), in NSCLC patients and discuss if the combinations of measured APPs could be exploited for assessing the success of immunotherapy with PD-1/PD-L1 checkpoint inhibitors.

## Materials and Methods

### Sample Collection, Characterization, and Preparation

Serum samples of patients with NSCLC were collected at the Thorax Clinic-Heidelberg and provided by Lung Biobank Heidelberg, a member of the accredited Tissue Bank of the National Center for Tumor Diseases (NCT) Heidelberg, the Biomaterial Bank Heidelberg, and the Biobank platform of the German Center for Lung Research (DZL). The use of biomaterial and data for this study was approved by the local ethics committee of the Medical Faculty Heidelberg (S-270/2001) and Hannover (9155_BO_K_2020). All patients included in the study signed informed consent and the study was performed according to the principles set out in the WMA Declaration of Helsinki. Investigated patient samples were part of a prospective cohort. The assembly of this cohort has started in 2018 and includes patients receiving checkpoint inhibitors at any therapy line. Up to now (May 2021), more than 400 patients with NSCLC have been included in the cohort, and these patients are currently followed up. At the time of analysis, 139 patients were available with a follow-up of at least 2 years. These patients were included in the study. Baseline blood sampling was performed up to 10 days prior to any immunotherapy. Blood was processed within 1 h after the blood draw. Serum aliquots were stored at −80°C until measurements. The patient cohort is described in **Table 1** and **Table S1**. All following measurements were performed using the whole cohort.

**Table 1:** Cohort description.

Parameter	n	(%)
Median age at diagnosis	63 (38–85)	
**Total**	**139**	**100**
Male	81	58
Female	58	42
**NSCLC Histology**		
Adeno	92	66
Squamous	32	23
NOS	9	6
other	4	3
**Clinical stage at time of IO treatment** **(8^th^ TNM edition)**		
III	15	11
IV	124	89
**Treatment**		
Pembrolizumab + chemotherapy	57	41
Pembrolizumab	35	25
Nivolumab	22	16
Atezolizumab	14	10
Durvalumab	10	7
Durvalumab + chemotherapy	1	1
**Treatment line immunotherapy**		
1^st^ line	72	52
2^nd^ line	57	41
3^rd^ line	6	4
4^th^ line	4	3

IO, Immuno-oncology; NSCLC, non-small cell lung cancer; NOS, not otherwise specified; TMN, tumor, node and metastasis.

### Measurements of Acute Phase Proteins

Serum concentrations of APPs were measured blindly using the nephelometric method (IMMAGE 800 Protein Chemistry Analyzer, Beckman Coulter Inc., CA, USA) in the Department of Genetics and Clinical Immunology at the National Institute of Tuberculosis and Lung Diseases, Warsaw. Analysis sensitivity for measured APPs was: ALB (22.2 mg/dL), AAT (10 mg/dL), AGP (35 mg/dL) hs-CRP (0.02 mg/dL), AT3 (5 mg/dL), CP (2 mg/dL), HP (5.83mg/dL) and A2M (40 mg/dL). All serum samples were analyzed at the same time, to control for testing variability. For HP, one data point is missing due to too the low sample amount. Plasma levels of ACT and SAA were measured by ELISA sandwich kit from BT Lab Bioassay Technology Laboratory (Shanghai, China) at 450 nm in a spectrophotometric reader Infinite M200 (Tecan, Austria). Assay sensitivity for ACT was 5.17 µg/ml and for SAA was 0.024 µg/ml. All standards and samples were analyzed in duplicates.

### Statistical Analyses

Data of serum measurements were visualized and statistically analysed with GraphPad Prism 9 (GraphPad Software, San Diego, California, USA) and SPSS 26.0 (IBM, Ehningen, Germany). Data were statistically analysed under REMARK criteria (12). Progression-free survival (PFS) time was calculated from the date of medication start until the last date of contact or progression of the disease under RECIST 1.1 criteria (24). Patients were included in analyses if PFS was > 30 days after therapy initiation. The cut-offs used for survival analyses were selected using the software tool “cutoff-finder” (25). “Survival” method of the tool was used. This method fits Cox proportional hazard models to the dichotomized variable and the survival variable. The optimal cutoff is defined as the point with the most significant (log-rank test) split. Hazard ratios (HRs) including 95% confidence intervals are calculated. Uni and multivariate survival analyses were performed using the Cox proportional hazards model. Visualization of survival data was performed according to Kaplan and Meier. Correlation analyses were performed using the nonparametric Spearman’s rank test. A correlation with r > 0.5 was considered as a reliable correlation. P-values are interpreted in a descriptive manner, no formal sample size calculation was done in this exploratory study.

## Results

### Correlation of APPs in Serum Samples of Patients With NSCLC

**Figure 1:**
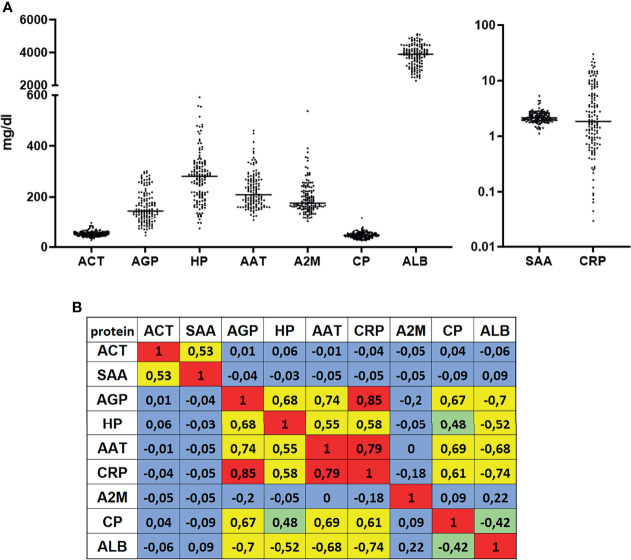
Serum concentrations and correlations of measured acute phase proteins. **(A)** Nine acute phase proteins were measured in serum of patients with NSCLC (n = 139) prior to a PD-1 or PD-L1 immunotherapy. **(B)** Spearman ranked correlation analyses of measured serum values. Values with r > 0.5 or r < -0.5 were considered as correlation. Colours indicate- red-strong correlation; yellow- reliable correlation; green-weak correlation; blue-no correlation.

To investigate serum APPs as predictive biomarkers for the immunotherapy response, levels of nine APPs (ACT, SAA, AGP, HP, AAT, CRP, A2M, CP and ALB) were measured in 139 patients with NSCLC prior to anti-PD-1 or anti-PD-L1 therapy (**Table 1** and **Table S1**). Serum concentrations of some investigated proteins, like AGP, HP, AAT, A2M, ALB and hs-CRP, varied strongly among patients (**Figure 1A**) while others, like ACT, CP, and SAA, were homogenous. To gain a better understanding of the relationships between analyzed APPs, we performed a correlation analysis (**Figure 1B**). Interestingly, we detected two sets of APPs, in which they correlated to each other. One set included AGP, HP, AAT, hs-CRP, CP, and ALB, with the highest correlations observed between hs-CRP and AGP (r = 0.85) and between hs-CRP and AAT (r = 0.79). Another set included ACT and SAA (r = 0.53) whereas A2M did not correlate with any of the measured APPs.

Most of the patients were diagnosed with an adenocarcinoma of the lung (66 %), at clinical stage IV (70 %). The patients initially received a variety of treatments while the major therapy was immuno-chemotherapy for 31 % of patients. The levels of APPs were analyzed at the start of immunotherapy in 124 patients at clinical stage IV and in 15 patients at stage III. As illustrated in **Figure S1**, APP levels did not differ significantly within the two stages. Regarding the immunotherapy drugs, 41 % of the patients received pembrolizumab + chemotherapy followed by pembrolizumab alone (25 %). We included patients from 1st to 4th line immunotherapy whereas approximately half of the patients (52 %) received the immunotherapy as 1st line treatment. The median progression-free survival of the cohort was 204 days (33 - 750 days).

### Some APPs Might Be Predictive for Immunotherapy Response

To get an idea of whether any of the measured proteins might be predictive for patient`s benefit receiving anti-PD-1 or anti-PD-L1 antibody immunotherapy, we performed a univariate Cox-regression analysis (**Table S2**). By using the software tool “cut-off finder”, optimal values of APPs were determined for the categorization of the patients (**Table S3**). Based on these cut-offs, two APPs (HP and CP) were highly predictive for the efficiency of immunotherapy when considered as single factors (**Table S2**). AGP, AAT, hsCRP and ALB failed to be highly robust markers after Bonferroni correction for multiple testing. Three other proteins, namely ACT, SAA and A2M, showed no significant predictive value.

**Figure 2:**
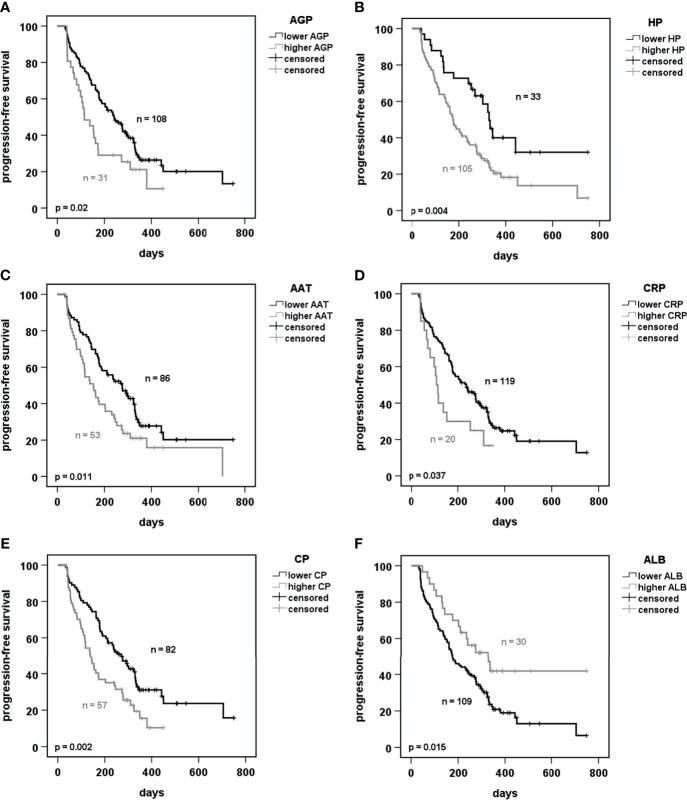
Prediction of progression-free survival of patients receiving PD-1 or PD-L1 immunotherapy dependent on acute phase proteins. **(A–F)** Kaplan-Meier curves of progression-free survival under immunotherapy in prediction to baseline serum concentrations of the indicated acute phase proteins. Cut-offs were calculated using the software tool “cutoff-finder” (25). p < 0.05 was considered as significant. AGP, alpha-1 acid glycoprotein; HP, haptoglobin; AAT, alpha1-antitrypsin; CRP, C-reactive protein; CP, ceruloplasmin; ALB, albumin.

Besides HP and CP, AGP, AAT, hs-CRP and ALB were also included for visualization by Kaplan Meier plots. The Cox regression model showed that these latter might be good predictors as well. In our cohort we observed that lower pre-therapy levels of AGP, HP, AAT, hs-CRP, and CP were predictive for significantly longer PFS (**Figures 2A–E**). By contrast, patients with higher ALB levels showed a longer PFS (**Figure 2F**). The predictive value of ACT and SAA was not significant while no optimal cut-off was discovered for A2M (**Figure S3**).

We also checked for any putative effect of anti-PD‐1 and PD‐L1 antibodies alone or when used with chemotherapy on the patient’s PFS (**Figure S2**). While a specific targeted therapy showed no influence (**Figure S2A**), line of therapy showed a borderline significance with the tendency for a benefit if immunotherapy was given as a 1st-line treatment (**Figure S2B**).

### A Signature of Two APPs Is Highly Predictive for an Immunotherapy Response in NSCLC

To further strengthen the power of APPs as biomarkers, we combined the cut-offs of HP and CP, since both proteins did not correlate (**Figure 1B**). We performed multivariate Cox regression analysis including the clinical parameters such as age, sex, cancer histology and clinical stage, line of treatment and target of immunotherapy (**Table S4**) and investigated every single factor for its robustness. We observed that HP and CP, when combined with clinical parameters, remained as significantly predictive markers. In fact, in all performed multivariate analyses, the serum concentration of the APPs was the factor with the highest predictive value for PFS. Interestingly, further multivariate analysis including HP and CP as well as the clinical parameters from **Table S4** showed that HP is the strongest independent marker (p = 0.007) (**Table S5**).

**Figure 3:**
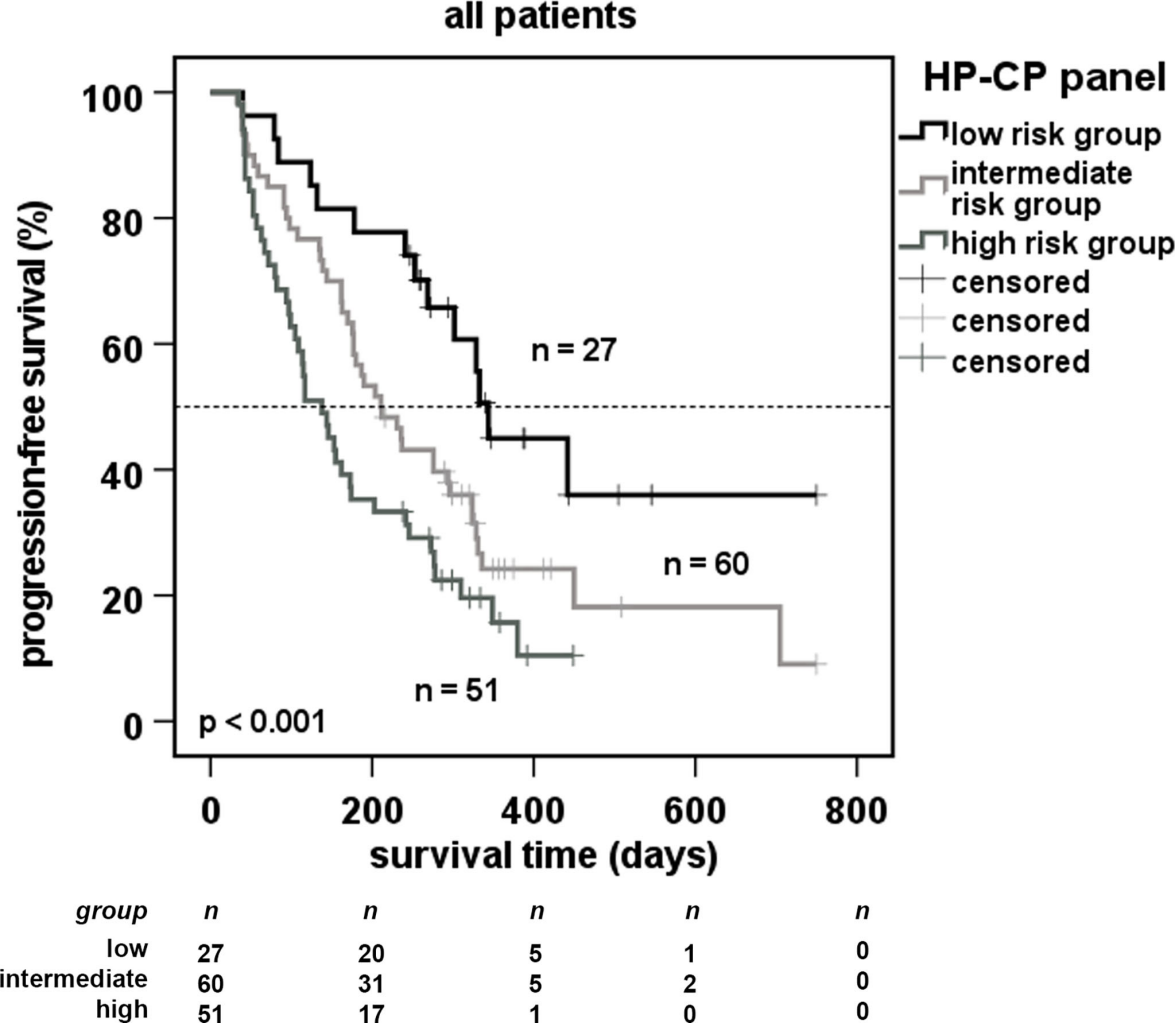
Predictive progression-free survival of patients using a 2-marker-panel of acute phase proteins. Cut-off values used in **Figure 2** were combined for robust markers of multivariate analysis (**Table S4**). Patients were separated in a low-risk group (serum values below cut-offs), a high-risk group (serum values above cut-offs) and an intermediate risk group (all other patients). Dotted line indicates the median survival of the cohort.

We next hypothetically divided all patients into 3 subgroups as having different risks for disease progression: i) a low risk (serum values of HP and CP below cut-offs from **Table S3**), ii) a high-risk (serum values above cut-offs from **Table S3**) and iii) an intermediate risk (serum values between) (**Figure 3**). The results demonstrate that patients from the low-risk group showed a highly increased PFS (upper curve), whereas high-risk patient group displayed a dramatic drop-down of progression-free survival. These results support a panel of two APPs as highly predictive for the success of immunotherapy in patients with NSCLC.

## Discussion

Currently approved criteria for patient selection to receive ICI monotherapy or ICI and chemotherapy combination do not reliably exclude non-responders or patients who develop treatment resistance over time (5). Therefore, individually adjusted treatment, based on predictive biomarkers of PFS, would be extremely helpful for enhancing therapeutic benefits and avoiding unnecessary costs of ICI treatment.

PD-L1 expression is typically used as a biomarker for PD-1/PD-L1 therapy since PD-1/PD-L1 signal pathway is a key target of ICIs. However, the predictive value of PD-L1 in NSCLC has been confirmed for the 1st but not for the 2nd line of treatment (26, 27). Besides PD-L1, another approved biomarker is microsatellite instability-high resulting from an impaired DNA mismatch repair. However, this is a rare condition and reflects an exceedingly small subpopulation of NSCLC patients, about 0,53% in lung adenocarcinoma, although different trials came with opposite conclusions (28).

Other biomarkers with a putative predictive value regarding the benefits of ICI therapy have also been examined. Tumour mutation burden (TMB), defined as the frequency of certain mutations within a tumour’s genes, has been considered as a very promising biomarker for dual ICI with significantly positive predictive value for better PFS and overall survival including patients negative for PD-L1 (29). Yet, its clinical application has been crashed by the inconsistency in the detection methods, and the lack of a standardized cut-off to define high TMB status. Likewise, the significance of tumour-infiltrating lymphocytes is difficult to implement as a biomarker for ICI response due to the technical constraints of assessment methods and biopsy material representativeness. The transcriptional signatures of immune responsiveness, like a ratio of CD8^+^ T versus T helper 1 cell cytokine mRNA, red blood cell distribution width (30), baseline serum sodium concentration (31), blood levels of prolactin (32) and positron emission tomography (33) were used to predict the PFS and overall survival in response to ICI therapy. Despite advances in new methodologies, routine measurement of specific tumour markers remains challenging because some of them are rapidly degraded, difficult to assay and/or masked by highly abundant blood proteins like ALB, AAT, or HP.

Different reports support an important role of inflammation in cancer cell proliferation, angiogenesis, and migration (34, 35). On the other hand, cancer cells themselves mediate systemic inflammation, which is coordinated by immune cells, cytokines/chemokines, and APPs, among others (36), and it is thought to contribute to cancer progression and cancer-related complications. Various APPs, specifically CRP, may reflect cancer-induced inflammatory processes (37). CRP has been shown to correlate with low levels of CD4+ T-cells, which play a key role in ICI-mediated antitumor immune response (38). Recent studies have indicated that pre-ICI therapy levels of CRP may represent a valuable prognostic marker in NSCLC (39–41).

In general, single APPs have been reported to have a potential value in guiding decision-making for patients undergoing chemotherapy and targeted ICI therapy. In the present study, we assessed whether a panel of APPs could have better prognostic and predictive value than single APPs and could be useful to predict a patient`s response to immunotherapy. Indeed, data from a retrospective cohort of patients harboring NSCLC treated with ICIs revealed that the pre-therapeutic levels of some APPs have good prognostic value. Lower pretherapeutic (baseline) concentrations of AGP, HP, AAT, hs-CRP, and CP but higher ALB were predictive for better PFS in our cohort. Finally, a panel of HP and CP preserved a powerful prognostic value even after multivariate Cox regression analysis combining APPs with clinical data. According to some reports, CRP/ALB ratio may have a prognostic value in cancer (42), unfortunately, we were not able to confirm this in our patient’s cohort. It is also important to notify that targeting therapy using anti-PD-1 or anti-PD-L1 alone or in combination with chemotherapy showed no influence on PFS time in our NSCLC cohort. However, a tendency towards a longer PFS was found if immunotherapy was given as a 1st-line treatment. The clinical decision of 1st line therapy is typically based on both, tumour and patients’, characteristics. Therefore, it remains incredibly important to decide when immunotherapy, using PD-1/PD-L1 immune checkpoint blockade as a 1st line therapy, is the right strategy for NSCLC patients (43).

Results from our exploratory cohort of 139 patients show that five APPs - AGP, HP, AAT, CP, and ALB - are promising biomarkers to predict the beneficial response of NSCLC patients to ICI therapy. Specifically, we show that a combination of HP and CP can highly improve our findings. HP, a marker of red blood cell destruction and a main hemoglobin-binding protein, which increases in many inflammatory diseases, including lung cancer. HP seems to be involved in the pathogenesis of tumors through innate/acquired immunity, effects on cell migration and angiogenesis, and glycolytic activity (44–46). Different authors found that circulating levels of HP increase with cancer stage and that HP is potentially useful in the clinical biomarker of lung cancer (47–49). CP is a well-known copper-binding protein, which has been associated with cancer development (50) and suggested as a useful biomarker for lung adenocarcinoma (51). Although CP is associated with tumor growth, invasiveness and prognosis in lung cancer patients, its biological role in tumorigenesis remains not fully understood. Other APPs, which are included in our analysis, have also been related to lung cancer and discussed as putative biomarkers. For example, in lung cancer patients’ serum level alterations were documented for AGP (52, 53), AAT (13), and ALB (54). A retrospective study evaluated the association of baseline serum ALB with the clinical outcome in a cohort of 457 patients with advanced-stage NSCLC treated with erlotinib, targeting the epidermal growth factor receptor. Remarkably, before the treatment initiation, low albumin was associated with poor outcomes of patients (55). Our findings are in line, as in our panel higher levels of ALB combined with lower levels of AGP, HP, AAT, and CP, are associated with better PFS of patients with NSCLC.

The changes in serum/plasma concentrations of APPs correlate with their increased hepatic synthesis (56) in response to tissue injury, inflammation, infection, or various malignancies. Although APPs are mainly produced in the liver, other organs/tissues may contribute like skin, lungs, kidneys, adipose tissue (57). In general, it has been thought that APPs are not tumour-derived and represent cancer epiphenomena rather than direct tumour-derived proteins. Recent proteomics studies profiling serum proteins of cancer and non-cancer individuals indicated that the altered levels of specific APPs can be observed in distinct types, subtypes, and stages of cancer (58). Moreover, others and we previously reported that cancer cells express and release APPs such as AAT or SAA (13).

The reproducibly of prognostic values of our suggested panel of APPs needs to be confirmed in an independent and larger sample set by us and other investigators. Since we are still increasing the cohort of NSCLC patients with immunotherapy, we will validate our findings in a larger cohort. If confirmed, this panel can be a valuable a predictive marker for NSCLC patient response to ICI therapy. In general, the analysis of APPs is a non-invasive and reliable method, available in all clinical chemistry laboratories, suggesting its high potential.

## Conclusion

A panel of two serum APPs, namely HP and CP, provides a clinically relevant pretherapeutic tool to predict the efficiency of PD-1/PD-L1 checkpoint inhibitor therapy as displayed in the progression-free survival of 139 NSCLC patients.

## Data Availability Statement

The raw data supporting the conclusions of this article will be made available by the authors, without undue reservation.

## Ethics Statement

The studies involving human participants were reviewed and approved by the local ethics committees of the Medical Faculty of the University of Heidelberg (S-270/2001) and of the Hannover Medical School (9155_BO_K_2020). The patients/participants provided their written informed consent to participate in this study.

## Author Contributions

MS: data analysis and presentation. AR, acute phase protein assay. SW: manuscript drafting. PC, TM, MT, and MM: providing patient cohort and clinical data. TW and JC-W: manuscript drafting. SJ: concept, manuscript preparation. All authors read and added comments to the manuscript. All authors contributed to the article and approved the submitted version.

## Funding

This research was funded by German Center for Lung Research (DZL), grant numbers 82DZL00402 and 82DZL002A1.

## Conflict of Interest

The authors declare that the research was conducted in the absence of any commercial or financial relationships that could be construed as a potential conflict of interest.

## Publisher’s Note

All claims expressed in this article are solely those of the authors and do not necessarily represent those of their affiliated organizations, or those of the publisher, the editors and the reviewers. Any product that may be evaluated in this article, or claim that may be made by its manufacturer, is not guaranteed or endorsed by the publisher.
