# Supplementary figures and images for: Acute Phase Proteins as Early Predictors for Immunotherapy Response in Advanced NSCLC: An Explorative Study
Source: Front Oncol. 2022 Jan 31;12:772076. doi: 10.3389/fonc.2022.772076 (PMC8841510; doi:10.3389/fonc.2022.772076)

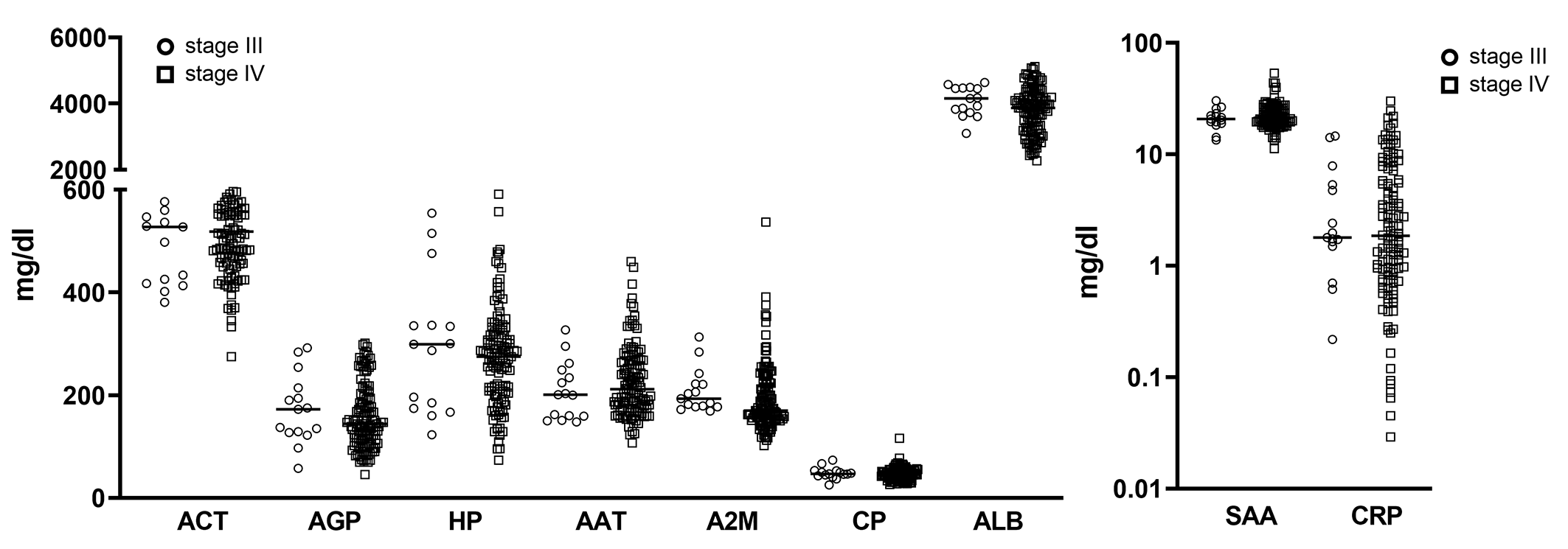

Supplement: Supplementary file 1 [file Image_1.tif]

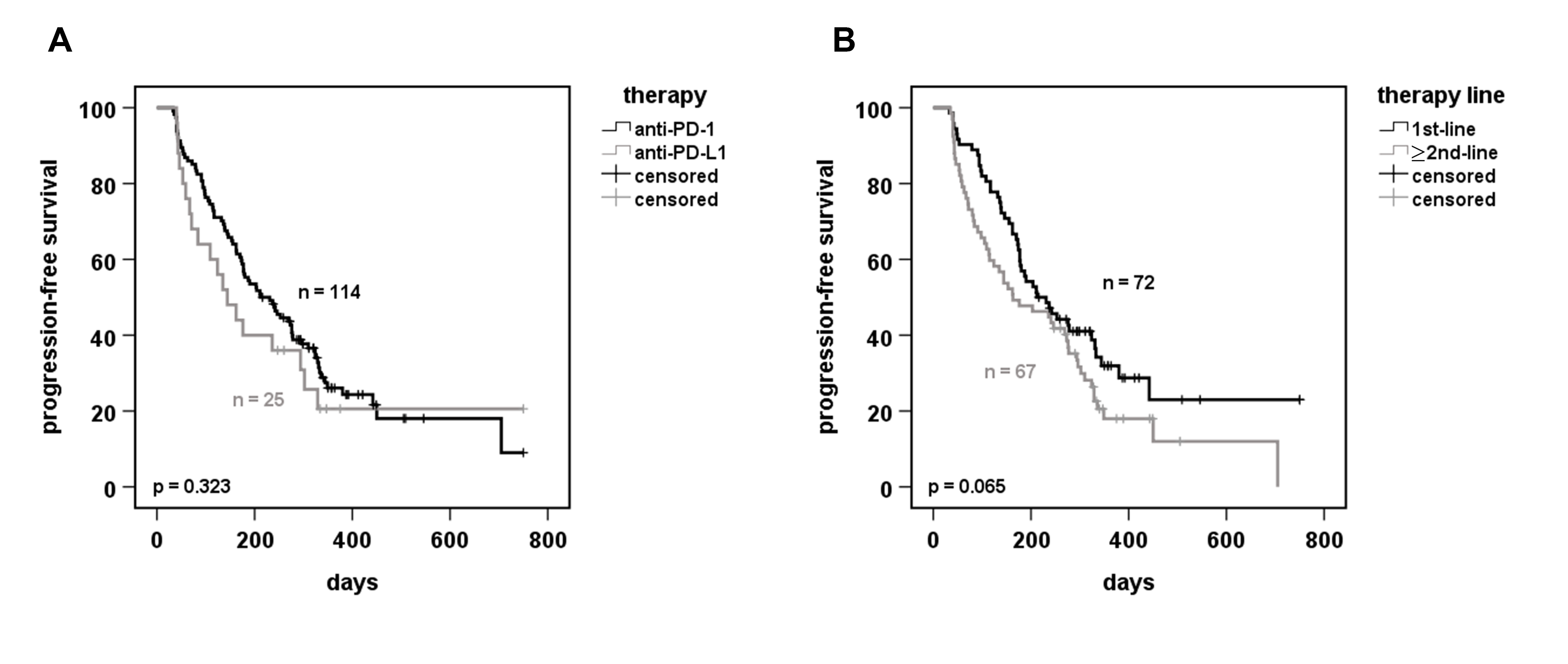

Supplement: Supplementary file 2 [file Image_2.tif]

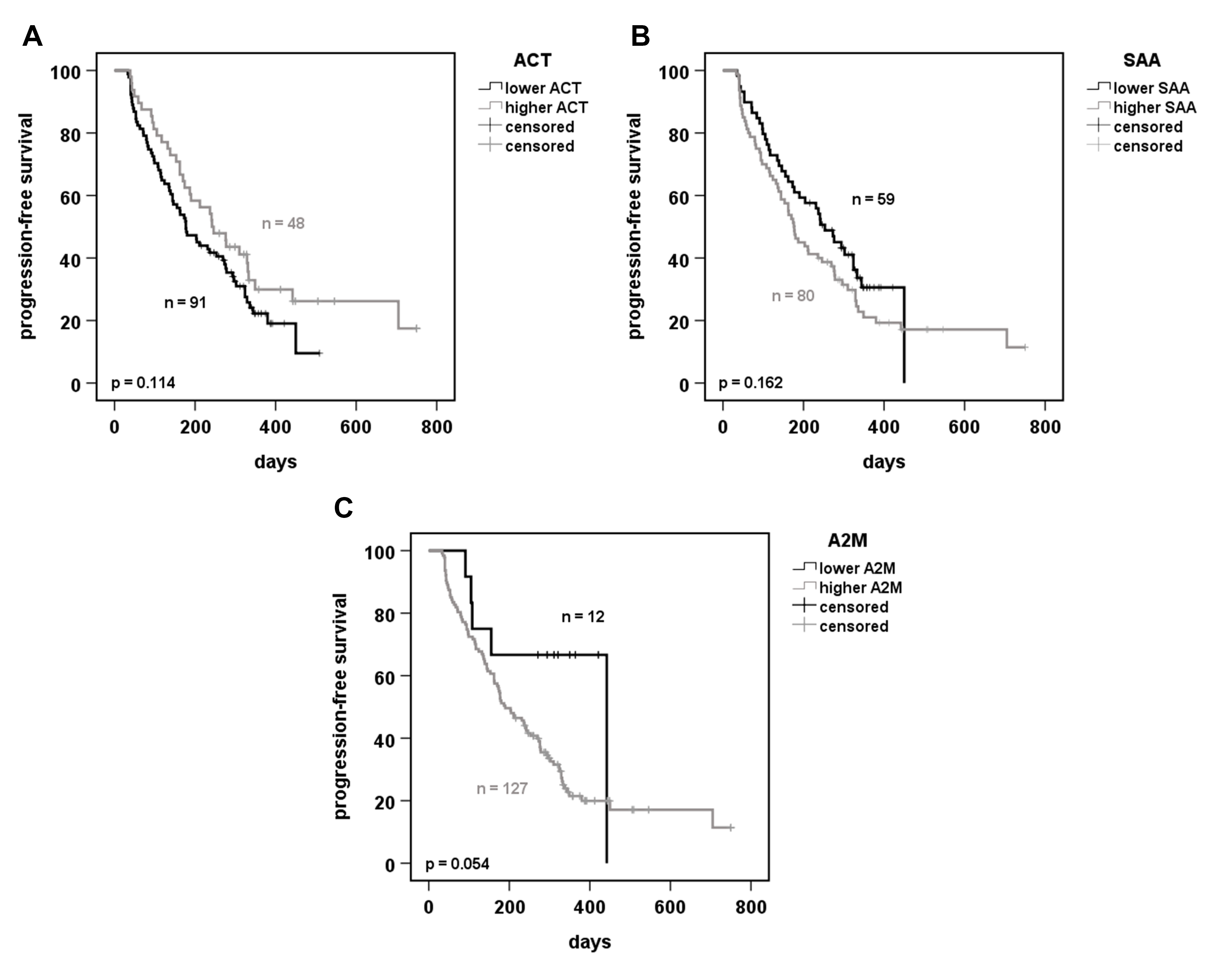

Supplement: Supplementary file 3 [file Image_3.tif]
