# Supplementary material for: Acute Phase Proteins as Early Predictors for Immunotherapy Response in Advanced NSCLC: An Explorative Study
Source: Front Oncol. 2022 Jan 31;12:772076. doi: 10.3389/fonc.2022.772076 (PMC8841510; doi:10.3389/fonc.2022.772076)
Supplement: Supplementary file 4 [file Table_1.docx]

| Table S1. Extended cohort description | | |
| --- | --- | --- |
| **Parameter** | **n** | (**%**) |
| Median age at diagnosis | 63 (38–85) |  |
| **Total** | **139** | **100** |
| Male | 81 | 58 |
| Female | 58 | 42 |
| **NSCLC Histology** |  |  |
| Adeno | 92 | 66 |
| Squamous | 32 | 23 |
| NOS | 9 | 6 |
| other | 4 | 3 |
| **Clinical stage at diagnosis**  **(8^th^ TNM edition)** |  |  |
| I | 7 | 5 |
| II | 5 | 4 |
| III | 30 | 22 |
| IV | 97 | 70 |
| **Initial Therapy** |  |  |
| Immunotherapy | 15 | 11 |
| Radiotherapy | 5 | 4 |
| Chemotherapy | 26 | 19 |
| Operation | 14 | 10 |
| Chemo-/Immunotherapy | 43 | 31 |
| Chemo-/Radiotherapy | 26 | 19 |
| Chemotherapy/Operation | 6 | 4 |
| other | 4 | 3 |
| **Clinical stage at time of IO treatment**  **(8^th^ TNM edition)** |  |  |
| III | 15 | 11 |
| IV | 124 | 89 |
| **Immunotherapy** |  |  |
| Pembrolizumab + chemotherapy | 57 | 41 |
| Pembrolizumab | 35 | 25 |
| Nivolumab | 22 | 16 |
| Atezolizumab | 14 | 10 |
| Durvalumab | 10 | 7 |
| Durvalumab + chemotherapy | 1 | 1 |
| **Treatment line immunotherapy** |  |  |
| 1^st^ line | 72 | 52 |
| 2^nd^ line | 57 | 41 |
| 3^rd^ line | 6 | 4 |
| 4^th^ line | 4 | 3 |

IO = Immuno-oncology, NSCLC = non-small cell lung cancer, NOS = not otherwise specified, TMN = tumor, node and metastasis
