# Supplementary material for: Acute Phase Proteins as Early Predictors for Immunotherapy Response in Advanced NSCLC: An Explorative Study
Source: Front Oncol. 2022 Jan 31;12:772076. doi: 10.3389/fonc.2022.772076 (PMC8841510; doi:10.3389/fonc.2022.772076)
Supplement: Supplementary file 5 [file Table_2.docx]

| Table S2. Univariate Cox-regression, corrected for multiple hypothesis testing (Bonferroni correction*) | | |
| --- | --- | --- |
| univariate analyses of progression-free survival (n = 139) | | |
| *variable* | ***HR (95% CI)*** | ***p*** |
| ACT (high vs. low) | 0.715 (0.470-1.087) | 0.114 |
| SAA | 1.330 (0.889-1.990) | 0.162 |
| AGP | 1.707 (1.083-2.691) | 0.02 |
| HP | 2.077 (1.245-3.466) | **0.004** |
| AAT | 1.659 (1.117-2.466) | 0.011 |
| CRP | 1.757 (1.025-3.013) | 0.037 |
| A2M | 2.357 (0.958-5.800) | 0.062 |
| CP | 1.831 (1.230-2.727) | **0.002** |
| ALB | 0.522 (0.306-0.891) | 0.015 |

HR = hazard ratio, CI = confidence interval, ACT = alpha1-antichymotrypsin, SAA = serum amyloid A, AGP = alpha-1 acid glycoprotein, HP = haptoglobin, AAT = alpha1-antitrypsin, CRP = C-reactive protein, A2M = alpha2-macroglobulin, CP = ceruloplasmin, ALB = albumin, *p-value <0.0055 was considered significant
