# Supplementary material for: Acute Phase Proteins as Early Predictors for Immunotherapy Response in Advanced NSCLC: An Explorative Study
Source: Front Oncol. 2022 Jan 31;12:772076. doi: 10.3389/fonc.2022.772076 (PMC8841510; doi:10.3389/fonc.2022.772076)
Supplement: Supplementary file 6 [file Table_3.docx]

| Table S3. Optimal cut-offs for survival analyses | | |
| --- | --- | --- |
| serum concentrations | | |
| *variable* | ***median (min-max) (mg/dl)*** | ***calculated cutoff (mg/dl)*** |
| ACT | 52.16 (27.49-95.15) | 55.8 |
| SAA | 2.14 (1.12-5.33) | 2.04 |
| AGP | 144 (45.4-301) | 210 |
| HP | 280.5 (73.3-591) | 201.8 |
| AAT | 208 (107-460) | 235.5 |
| CRP | 1.84 (0.03-30) | 10.1 |
| A2M | 175 (102-537) | 132.5 |
| CP | 45.6 (25.2-116) | 48.5 |
| ALB | 3890 (2270-5120) | 4420 |

HR = hazard ratio, CI = confidence interval, ACT = alpha1-antichymotrypsin, SAA = serum amyloid A, AGP = alpha-1 acid glycoprotein, HP = haptoglobin, AAT = alpha1-antitrypsin, CRP = C-reactive protein, A2M = alpha2-macroglobulin, CP = ceruloplasmin, ALB = albumin
