# Supplementary material for: Acute Phase Proteins as Early Predictors for Immunotherapy Response in Advanced NSCLC: An Explorative Study
Source: Front Oncol. 2022 Jan 31;12:772076. doi: 10.3389/fonc.2022.772076 (PMC8841510; doi:10.3389/fonc.2022.772076)
Supplement: Supplementary file 7 [file Table_4.docx]

| Table S4. Multivariate Cox regression, corrected for multiple hypothesis testing (Bonferroni correction*) | | |
| --- | --- | --- |
| multivariate analyses of progression-free survival | | |
| *variable* | ***HR (95% CI)*** | ***p*** |
| Age at diagnosis | 1.015 (0.992-1.039) | 0.211 |
| Sex (female vs. male) | 1.106 (0.740-1.653) | 0.623 |
| Histology | 0.913 (0.746-1.118) | 0.378 |
| Clinical stage at diagnosis | 1.072 (0.796-1.445) | 0.647 |
| Line of immunotherapy | 1.424 (0.928-2.183) | 0.105 |
| Target treatment (PD-L1 vs. PD-1) | 1.179 (0.687-2.021) | 0.551 |
| AGP (high vs. low) | 1.692 (1.064-2.690) | 0.026 |
| *variable* | ***HR (95% CI)*** | ***p*** |
| Age at diagnosis | 1.022 (0.998-1.047) | 0.076 |
| Sex (female vs. male) | 1.144 (0.763-1.717) | 0.515 |
| Histology | 0.824 (0.649-1.045) | 0.111 |
| Clinical stage | 1.065 (0.799-1.420) | 0.667 |
| Line of immunotherapy | 1.344 (0.873-2.069) | 0.179 |
| Target treatment (PD-L1 vs. PD-1) | 1.612 (0.912-2.851) | 0.1 |
| HP (high vs. low) | 2.493 (1.448-4.291) | **<0.001** |
| *variable* | ***HR (95% CI)*** | ***p*** |
| Age at diagnosis | 1.012 (0.988-1.037) | 0.327 |
| Sex (female vs. male) | 1.144 (0.763-1.716) | 0.514 |
| Histology | 0.919 (0.752-1.124) | 0.413 |
| Clinical stage | 1.033 (0.770-1.386) | 0.828 |
| Line of immunotherapy | 1.412 (0.922-2.161) | 0.113 |
| Target treatment (PD-L1 vs. PD-1) | 1.297 (0.749-2.247) | 0.354 |
| AAT (high vs. low) | 1.684 (1.107-2.561) | 0.015 |
| *variable* | ***HR (95% CI)*** | ***p*** |
| Age at diagnosis | 1.015 (0.992-1.038) | 0.215 |
| Sex (female vs. male) | 1.070 (0.719-1.594) | 0.738 |
| Histology | 0.914 (0.749-1.115) | 0.376 |
| Clinical stage | 1.064 (0.795-1.423) | 0.679 |
| Line of immunotherapy | 1.418 (0.924-2.174) | 0.11 |
| Target treatment (PD-L1 vs. PD-1) | 1.155 (0.672-1.984) | 0.603 |
| CRP (high vs. low) | 1.635 (0.947-2.823) | 0.078 |
| *variable* | ***HR (95% CI)*** | ***p*** |
| Age at diagnosis | 1.021 (0.997-1.045) | 0.093 |
| Sex (female vs. male) | 1.039 (0.698-1.545) | 0.852 |
| Histology | 0.939 (0.771-1.144) | 0.533 |
| Clinical stage | 0.988 (0.732-1.335) | 0.940 |
| Line of immunotherapy | 1.383 (0.899-2.128) | 0.14 |
| Target treatment (PD-L1 vs. PD-1) | 1.226 (0.713-2.108) | 0.462 |
| CP (high vs. low) | 1.926 (1.276-2.907) | **0.002** |
| *variable* | ***HR (95% CI)*** | ***p*** |
| Age at diagnosis | 1.010 (0.986-1.034) | 0.41 |
| Sex (female vs. male) | 1.191 (0.792-1.793) | 0.401 |
| Histology | 0.903 (0.736-1.106) | 0.324 |
| Clinical stage | 0.984 (0.74-1.309) | 0.912 |
| Line of immunotherapy | 1.363 (0.882-2.107) | 0.164 |
| Target treatment (PD-L1 vs. PD-1) | 1.313 (0.753-2.290) | 0.337 |
| ALB (high vs. low) | 0.498 (0.280-0.884) | 0.017 |

HR = hazard ratio, CI = confidence interval, AGP = alpha-1 acid glycoprotein, HP = haptoglobin, AAT = alpha1-antitrypsin, CRP = C-reactive protein, CP = ceruloplasmin, ALB = albumin. * A p-value < 0.0071 was considered significant.
