# Supplementary material for: Acute Phase Proteins as Early Predictors for Immunotherapy Response in Advanced NSCLC: An Explorative Study
Source: Front Oncol. 2022 Jan 31;12:772076. doi: 10.3389/fonc.2022.772076 (PMC8841510; doi:10.3389/fonc.2022.772076)
Supplement: Supplementary file 8 [file Table_5.docx]

| Table S5. Multivariate Cox regression, corrected for multiple hypothesis testing (Bonferroni correction*) | | |
| --- | --- | --- |
| multivariate analyses of progression-free survival | | |
| *variable* | ***HR (95% CI)*** | ***p*** |
| Age at diagnosis | 1.022 (0.997-1.048) | 0.086 |
| Sex (female vs. male) | 1.137 (0.758-1.704) | 0.536 |
| Histology | 0.836 (0.657-1.064) | 0.145 |
| Clinical stage | 0.988 (0.739-1.320) | 0.935 |
| Line of immunotherapy | 1.102 (0.857-1.417) | 0.449 |
| Target (PD-L1 vs. PD-1) | 1.723 (0.998-2.975) | 0.051 |
| HP | 2.164 (1.232-3.803) | 0.007 |
| CP | 1.631 (1.069-2.489) | 0.023 |

HR = hazard ratio, CI = confidence interval, HP = haptoglobin, CP = ceruloplasmin *A p-value < 0.0063 was considered significant.
